# Association of 25-Hydroxyvitamin D Deficiency in Pediatric Epileptic Patients

**Published:** 2017

**Authors:** Jaydip Ray CHAUDHURI, Kandadai Rukmini MRIDULA, Chakrala RATHNAKISHORE, Banda BALARAJU, VCS Srinivasarao BANDARU

**Affiliations:** 1 Department of Neurology, Yashoda hospital, Hyderabad, India; 2 Department of Neurology, Nizam’s Institute of Medical Sciences, Hyderabad, India; 3 Department of Neurology, Citi Neuro Center, Hyderabad, India; 4 Department of Medicine, Yashoda hospital, Hyderabad, India; 5 Department of Clinical Research, Yashoda hospital, Hyderabad, India

**Keywords:** Epilepsy, 25-hydroxyvitamin D, Alkaline Phosphatase, Calcium, Anticonvulsants Carbamazepine, Valproic Acid, Indian patients

## Abstract

**Objective:**

Epilepsy is a chronic neurological disorder requiring long-term therapy using antiepileptic medications. Reports have incriminated long-term antiepileptic drugs use in deficiency of vitamin D and bone diseases in all age groups. We aimed to investigate the association between serum 25-hydroxyvitamin D levels and pediatric epilepsy in Indian patients.

**Materials & Methods:**

We prospectively recruited 100 pediatric epilepsy patients, on monotherapy for minimum one-year duration, and 50 age and sex matched controls. This study was carried out at Yashoda Hospital, India from 2011-2014. All cases and controls underwent tests for serum 25-hydroxyvitamin D, alkaline phosphatase, serum calcium and phosphorus levels.

**Results:**

Patients with 25-hydroxyvitamin D deficiency were significantly higher among cases (45%) than controls (24%). Mean alkaline phosphatase was significantly higher in cases and mean serum calcium was significantly lower (8.3±1.5) in cases. Amongst antiepileptic drugs, carbamazepine and sodium valproate were significantly associated with 25-hydroxyvitamin D deficiency. Risk of vitamin D deficiency was highest with sodium valproate usage (odds:4.0;95%CI 1.4-11.6) followed by carbamazepine use (odds: 2.7; 95%CI 1.0-6.8). After adjustment using multiple logistic regression, antiepileptic drugs showed independent association with 25-hydroxyvitamin D deficiency (odds:2.2;95%CI 0.9-4.5).

**Conclusion:**

25-hydroxyvitamin D deficiency was significantly associated with use of carbamazepine and sodium valproate in pediatric epilepsy.

## Introduction

Epilepsy is a common neurological disorder that affects all age groups. Globally, around 50 million people are affected with epilepsy, which corresponds to 0.5% of the global burden of all diseases, in developing countries (1). In India, more than 10 million people suffer with seizures and prevalence of epilepsy is one percent in our population (2). The frequency rates for children vary from 2.0 to 22.2 per 1000 (3) and nearly 50% of all epileptics suffer from their childhood (4).

Epilepsy patients require longtime antiepileptic drugs (AEDs). Long-term usage of AEDs is associated with abnormal bone mineral metabolism, osteoporosis and an increased risk of fracture (5). The main mechanism seems to be centered on vitamin D deficiency caused by AEDs in epilepsy patients (6).

The structure of vitamin D is a 9,10 -seco steroid and two types are dominantly present in humans (vitamin D3 and vitamin D2). In circulation, vitamin D exists as serum 25-hydroxyvitamin D and this serves as a marker of vitamin D levels in the body. Vitamin D plays a vital role in maintaining a balance between phosphorus and calcium levels as well as metabolism. Vitamin D deficiency can cause reduced bone mineralization and bone fractures (7).

The aim of the present study was to investigate the circulating blood levels of serum 25-hydroxyvitamin D in pediatric epilepsy patients who were on AEDs. Very limited data is available from the Indian Subcontinent. 

## Material and Methods

We selected 100 consecutive pediatric epilepsy cases, with age below 18 yr and 50 age and sex matched controls. This study was carried out at Department of Neurology, Yashoda Hospital, Hyderabad, India from January 2011 to December 2014. This study was approved by Institutional Ethics Committee. Informed consent was taken from the subjects.

**Definition of cases and controls**

Epilepsy is a disease of the brain, which results in predisposition to have epileptic seizures. Epilepsy is diagnosed when there is occurrence of at least two unprovoked seizures, occurring at least 24 hours apart (8). Controls were recruited from healthy subjects (children of staff or volunteers) from same hospital. 

**Inclusion and exclusion criteria of case controls**

Patients who gave history of two attacks of seizures at least 24 hours apart in their life and were on monotherapy with antiepileptic drugs for minimum one year were included. Healthy controls without any history of seizures, rickets or other bone related disorders were included in the study. Patients who were on two or more AEDs, taking vitamin D supplements, suffering from other systemic diseases or with neurological deficits and controlswith any history of illness or taking vitamin D supplements were excluded in the study.

Standardized questions were adapted from behavioral risk factors questionnaire by the Centers for Disease Control and Prevention (CDC) (9). Patients’ data were collected through face-to-face interviews from patients’ parents among cases and control subjects by a senior epileptologist. Patient medical history, type of AEDs, physical and neurological examination was done by a senior neurologist. All cases and controls underwent estimation of serum alkaline phosphatase, serum calcium and serum phosphorus.

**Assessment of 25-hydroxyvitamin D**

For measurement of 25-hydroxyvitamin D, 5 ml blood was collected from both cases and controls. We used chemiluminescent microparticle immunoassay (CMIA) with automated instruments for assessment of 25-hydroxyvitamin D. We considered serum levels of 25 hydroxyvitamin D < 20 ng/mL as deficiency and >20.1 ng/mL as sufficient (7).

**Statistical analysis**

Statistical analysis was applied by SPSS ver. 15.0 (Chicago, IL, USA) software. Mean and standard deviation (SD) were estimated for various variables. 

Categorical variables were expressed as proportions and chi-square test was used to study the association in proportions. The student ‘t’ test was used to test the differences in continuous variables. We performed multiple logistic regression analysis before and after adjustment for various AEDs (Carbamazepine, clobazam, clonazepam, lamotrigine, phenobarbital, sodium valproate, topiramate). All tests were two sided and P value <0.05 was considered statistically significant.

## Results

We noted boys constituted 60% in both cases and controls. Mean age was 14 yrs in cases and 14.5 yrs in controls with age range of 8-18 yrs. We found significantly higher prevalence of deficiency of 25-hydroxyvitamin D, (P<0.0001) mean alkaline phosphatase (P<0.0001) and serum calcium (P=0.01) in cases compared to controls (Table 1).

Mean serum alkaline phosphatase (P=0.001), serum calcium (P=0.0006) and mean duration of taking AEDs (P<0.0001) were significantly higher among 25-hydroxyvitamin D deficient cases compared to cases with normal 25-hydroxyvitamin D levels (Table 2). 

**Fig 1:**
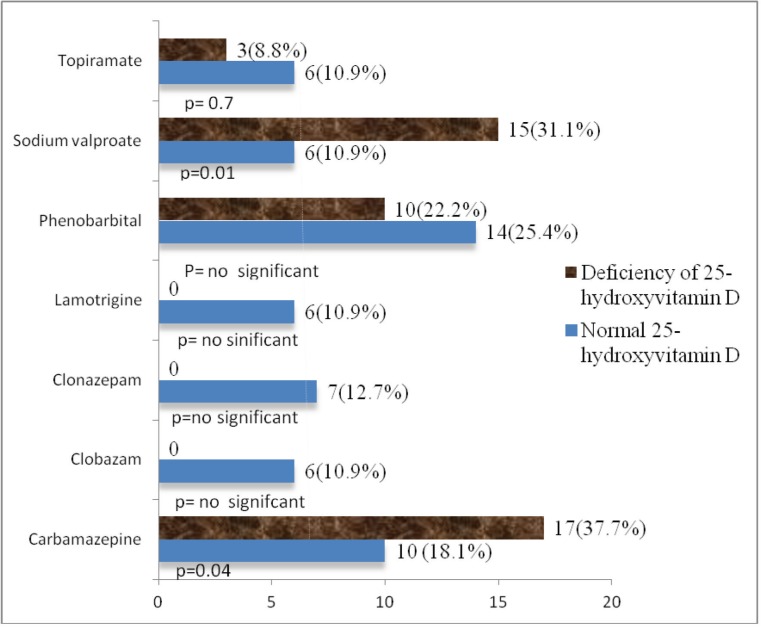
Different between normal and deficiency of 25-hydroxyvitmain D with various antiepileptic drugs

Regarding AEDs, the percentage taking carbamazepine was 27(27%), clobazam 6(6%), clonazepam 7(7%), lamotrigine 6(%), phenobarbital 24(24%), sodium valproate 21(21%) and topiramate 9(9%). Significantly higher prevalence of 25-hydroxyvitamin D deficiency was noted with carbamazepine (P=0.04) and sodium valproate (P=0.01) usage (Figure 1).

On odds ratio analysis, we established independent association of 25-hydroxyvitamin D deficiency with AEDs use. Carbamazepine (odds:2.7;95%CI:1.0-6.8) and sodium valproate (odds:4.0;95%CI:1.4-11.6) were associated more in comparison to phenobarbital (odds:2.0; 95%CI:0.8-5.1), and topiramate (odds:0.5;95%CI:0.1-2.4) use (Table 3).

After adjustment with multiple logistic regression analysis, antiepileptic drugs use was independently associated with deficiency of 25-hydroxyvitamin D in pediatric epilepsy patients (Table 4).

## Discussion

In our study, 25-hydroxyvitamin D (≤20 ng/mL) deficiency was significantly higher among epileptics (44%) compared to control subjects (20%), which is a constant finding noted by similar studies (6, 10, 11). Twenty five percent of children with epilepsy had deficiency of 25-hydroxyvitamin D (10). Serum 25-hydroxyvitamin D deficiency was 75% of children with epilepsy (12).

We also found significantly lower mean calcium levels in epileptic patients compared to controls (P= 0.01), our observation was advocated by others (12-14). 

The mechanism underlying low levels of calcium in epileptics may be multifactorial. AEDs are associated with alterations in bone metabolism and phosphate concentration and thus a change in calcium homeostasis in the body (15). However, some studies have found no significant association of calcium levels with epilepsy (16-18).

We noted mean alkaline phosphatase levels were significantly higher in epileptic patients compared to controls (P<0.0001) similar to other studies (4, 15, 16). 

Elevated alkaline phosphatase levels are associated with liver or bone disease. In the present study, high alkaline phosphatase levels are indicative of high bone turnover rather than liver disease as other hepatic parameters are normal.

**Table1:** Baseline Characteristics

**Parameters**	**Cases** **(n=100)**	**Controls** **(n=50)**	***P*** ** value**
**Male child**	60(60%)	30(60%)	0.8
**Female child**	40(40%)	20(40%)	0.8
**Mean age (years)**	14±2.2	14.5±2.5	0.8
**Age range (years)**	8-18	8-18	
**Range of 25-hydroxyvitamin D value**	9-25	15-30	
**25-hydroxyvitamin D deficiency**	45(45%)	12(24%)	=0.004
**Mean 25-hydroxyvitamin D deficiency**	18.3±6.2	27.7±3.9	<0.0001
**Mean alkaline Phosphatase**	564.3±157.3	386±294.7	<0.0001
**Mean serum calcium**	8.3±1.5	8.9±1.2	=0.01
**Mean serum phosphorus**	2.8±0.1	2.7±0.9	0.2

**Table 2:** Comparison between 25-Hydroxyvitamin D Deficient and Normal 25-Hydroxyvitamin D levels in Cases

**Types of antiepileptic drugs**	**Normal 25-hydroxyvitamin D** **(n=55)**	**Deficiency of** **25-hydroxyvitamin D** **(n=45)**	***P*** ** value**
**Male**	32(58.1%)	27(60%)	0.9
**Female**	23(41.8%)	18(40%)	0.9
**Mean age (years)**	13.7±2.4	14.1±1.9	0.3
**Age range (years)**	8-18	9-18	
**Mean Elevated alkaline Phosphatase**	523.9±139.8	619.7±159.8	=0.001
**Mean serum calcium**	8.7±1.4	7.7±1.4	=0.0006
**Mean serum phosphorus**	2.9±0.5	2.8±0.6	0.3
**Mean length of taking AEDs in months**	25.2±10.5	40.4±12.8.	<0.0001

The present study established that mean 25-hydroxyvitamin D levels was significantly lower in cases (18.3±6.2) compared to controls (27.7±3.9) (P<0.0001). In similar a study, the mean level of 25-hydroxyvitamin D was lower among cases (28.79 ± 33.85) in contrast to controls (mean 47.62 ± 46.16) (19). However, some studies have found no relationship between deficiency of 25-hydroxyvitamin D and epilepsy (15, 16, 20).

We noted that among epileptics, elevated alkaline phosphatase and low serum calcium were significantly associated with deficiency of 25-hydroxyvitamin D (P=0.0001), accounted by others (12-14, 21). 

Thus in our study the main pathogenetic mechanism seems to be based on reduced active levels of vitamin D, possibly caused by induction of hepatic cytochrome P450 enzymes by AEDs, leading to its conversion to inactive metabolites in the liver microsomes. Hypocalcemia can be due to decreased absorption from the gut secondary to the state of hypovitaminosis D. This may then trigger an increase in circulating parathyroid hormone. The secondary hyperparathyroidism then leads to an increased bone turnover leading to increased serum alkaline phosphatase levels (21).

Apart from its action on vitamin D, AEDs also have had an independent association with both increased parathormone levels and increased bone turnover (22). 

In this study, most commonly used antiepileptic drug was carbamazepine in 27%, phenobarbital in 24%, sodium valproate in 21%, topiramate in 9%, clonazepam 7%, clobazam 6% and lamotrigine in 6% of patients. 

Our study established that 25-hydroxyvitamin D deficiency was significantly associated with carbamazepine (37.7%) and sodium valproate (31.1%) usage; similar studies have advocated our findings (4, 6, 23, 24). Misra et al. found 25-hydroxyvitamin D deficiency in 21.7% of patients on carbamazepine (4).

A higher prevalence of 25-hydroxyvitamin deficiency (50%) with carbamazepine usage was noted (25). 

Verrotti et al. established an independent association between 25-hydroxyvitamin D deficiency and AEDs usage (26). However, some studies did not find any significant association of carbamazepine and sodium valproate usage with deficiency of 25-hydroxyvitamin D (27).

The present study showed no significant association between 25-hydroxyvitamin D deficiency with long-term administration of phenobarbital and topiramate. However, some studies have found deficiency of 25-hydroxyvitamin D with phenobarbital and topiramate (28, 29).

**Table3:** Odds Ratio with Antiepileptic Medication

**Antiepileptic drugs**	**Odds ratio**	**95% CI**
**Carbamazepine**	2.7	1.0-6.8
**Phenobarbital**	2.0	0.8-5.1
**Sodium valproate**	4.0	1.4-11.6
**Topiramate**	0.5	0.1-2.4

**Table 4:** Predictors of Deficiency of 25-Hydroxyvitamin D in Pediatric Epilepsy

	**Before adjustment**		**After adjustment**	
**Antiepileptic drugs**	Odds ratio	95% CI	Odds ratio	95% CI
**Any medication**	2.5	1.1-5.5	2.2	1.0-4.5
**Individual medication**				
**Carbamazepine**	2.7	1.0-6.8	*	*
**Phenobarbital**	2.0	0.8-5.1	*	*
**Sodium valproate**	4.0	1.4-11.6	*	*
**Topiramate**	0.5	0.1-2.4	*	*

* Number of patients insufficient for statistical analysis

Vitamin D inactivation by AEDs occurs mainly by induction of hepatic enzymes and by their activation of pregnane X receptor (PXR) and steroid and xenobiotic receptors (SXR) (22, 30). Activation of vitamin D (D2 and D3) occurs initially in the liver where they are hydroxylated to 25(OH) D by vitamin D hydroxylase CYP27A. The antiepileptic drug binds and activates SXR. This complex binds to RXR, which then activates the 24-hydroxylase enzyme by interacting with its vitamin D responsive element (31, 32). This enzyme mediates the removal of 25-hydroxyl group from both 25-hydroxyvitamin D and 1,25 dihydroxy vitamin D. 

This accelerated inactivation of vitamin D causes a cascade of events to adapt to the progressive insufficiency leading to secondary hyperparathyroidism. Further hypovitaminosis D results in decreased absorption of calcium from the gut. It has a detrimental effect on bone mineralization and metabolism. 

The above mechanism have been attributed to AEDs which are inducers of cytochrome P450 enzyme system (phenobarbitol, phenytoin and carbamazepine) (33).The other mechanism postulated include reduced intestinal calcium absorption (phenytoin) (34), impaired response to parathyroid hormone (phenobarbitone and phenytoin), (35) hypovitaminosis K (phenytoin) (36), and calcitonin deficiency (37). The exact mechanism by which sodium valproate causes similar bone mineral metabolism abnormalities is not clear but it may be mediated by a different hepatic enzyme inhibition (38). Multi-drug therapy is associated with high risk of bone mineral metabolism abnormalities than monotherapy (24). 

There has been a lot of debate on whether the enzyme inducing properties of AEDs are to blame. Initial studies reported an association of reduced bone mineral density and increased fracture risk with mainly enzyme inducing AEDs (EIAEDs) (39, 40). However, recent studies have found no difference between EIAEDs and Non-EIAEDs in their action on 25-hydroxyvitamin D status (41). There was a similar risk of developing vitamin D deficiency with both EIAEDs and Non-EIAEDs (42). In our study too, both EIAEDs (carbamazepine (odds 2.7;95%CI:1-0-6.8)) and Non- EIAEDs (sodium valproate (odds 4.0;95%CI:1-4-11.6)) were significantly associated with deficiency of 25-hydroxyvitamin D.

The duration of treatment especially long term usage of AEDs was significantly associated with 25-hydroxyvitamin D deficiency (43-46). We found that epileptics with deficiency of 25-hydroxyvitamin D had significantly longer duration of treatment (mean during of treatment 40.4±12.8 in months) compared to those with normal 25-hydroxyvitamin D (25.2±10.5 months). 

However, some studies have found no correlation between 25-hydroxyvitamin D deficiency and duration of treatment with AEDs (15, 20). The exact duration of AEDs, which leads to vitamin D deficiency, is not clear. 

In a study by Cansu et al, vitamin D levels significantly decreased after 18 months of taking AEDs (43). In another study, 49% acquired vitamin D3 insufficiency within 3 months of AEDs (45). Farhat et al, noted that exposure to AEDs for more than six month leads to vitamin D deficiency in 35% (46). In our study the cohort of epileptics with normal vitamin D had a mean exposure time of 25.2 months which was considerably longer than the previous data, this may be because Indians have more exposure to sunlight. India is a tropical country and the study was conducted in South India in a city with the temperature during the day of 20-30 ^o^C with only a 10 ^o^C variation between winter and summer.

This study reemphasizes the negative action of AEDs on bone mineral metabolism and is consistent with current worldwide literature (24). Vitamin D supplementation may help in preventing these complications. Several randomized controlled studies have shown a beneficial effect of vitamin D therapy with AEDs in children; however, the information regarding duration of therapy, role of diet/exercise and the role of monitoring to vitamin D levels are still lacking (47). They are currently no accepted guidelines for preventing and treating diseases of bone metabolism and vitamin D deficiency in epilepsy. Some authors suggest that doses as high as 50,000 IU of vitamin D monthly would be needed to normalize vitamin D levels for patients with epilepsy (48). Others have recommended a supplementation dose of 400-4000 IU/day of 25-hydroxyvitamin D for treating these changes. For prophylaxis, two studies have recommended 600- 2000IU/ day of vitamin D for all epileptics as soon as they are started on AEDs and for treating osteomalacia they have recommended a dosage of 5000-15000IU/day of vitamin D (49, 50). 

Most practicing neurologists do not consider this aspect while treating their epileptic patients. Prophylactic calcium or vitamin D supplementation is prescribed by only 9% of pediatric neurologists and 37% of neurologists, along with AEDs in epileptic patients (51). 

Our study had some limitations, the main inadequacy was that 25-hydroxyvitamin D levels were not measured before starting AEDs and hence we cannot categorically attribute the levels to AEDs use. Second drawback was that we did not assess bone density in both cases and controls due to both financial reasons and lack of standardized reference range for Indian children. Third, as our sample size was small, we were unable to perform multiple logistic regression analysis to study the association between individual AEDs and 25-hydroxyvitamin D levels. Strengths of our study were that we recruited cases and controls from the same hospital thus reducing difference based on ethnicity, social customs and socioeconomic status. 

In conclusion, this study is further reemphasis the need to create more bone health awareness among epileptic patients and health care providers. We believe that all epileptic patients should be counselled regarding calcium and vitamin D intake, exposure to sunlight and physical activity, especially before initiation of AEDs. 

There should be precautionary assessment especially when starting on AEDs like carbamazepine and sodium valproate, which have an increased risk of impairing bone mineral metabolism. Further multicentric prospective studies are required to evaluate the role and formulate clear guidelines on prophylactic vitamin D supplementation and other measured needed for prevention and treatment of impaired bone metabolism in epilepsy.
